# Hemodynamic impact of the connection to continuous renal replacement therapy in critically ill children

**DOI:** 10.1007/s00467-018-4047-7

**Published:** 2018-08-15

**Authors:** Sarah Fernández, Maria José Santiago, Rafael González, Javier Urbano, Jorge López, Maria José Solana, Amelia Sánchez, Jimena del Castillo, Jesús López-Herce

**Affiliations:** 1 Paediatric Intensive Care Department, Hospital General Universitario Gregorio Marañón, Instituto de Investigación del Hospital General Universitario Gregorio Marañón, Dr Castelo 47, 28009 Madrid, Spain; 2 Complutense University of Madrid, Madrid, Spain; 3 Spanish Health Institute Carlos III Maternal, Child Health and Development Network, Madrid, Spain

**Keywords:** Acute kidney injury, Renal failure, CRRT, Complications, Children

## Abstract

**Background:**

Continuous renal replacement therapy (CRRT) is the treatment of choice for critically ill children with acute kidney injury. Hypotension after starting CRRT is frequent but very few studies have analyzed its incidence and clinical relevance.

**Methods:**

A prospective, observational study was performed including critically ill children treated with CRRT between 2010 and 2014. Hemodynamic data and connection characteristics were collected before, during, and 60 min after CRRT circuit connection. Hypotension with the connection was defined as a decrease in > 20% of the mean arterial pressure from baseline or when intravenous fluid resuscitation or an increase in vasopressors was required.

**Results:**

One hundred sixty-one connections in 36 children (median age 18.8 months) were analyzed. Twenty-eight patients (77.8%) were in the postoperative period of cardiac surgery, 94% had mechanical ventilation, and 86.1% had vasopressors. The heparinized circuit priming solution was discarded in 8.7% and infused to the patient in 18% of the connections. The circuit was re-primed in the remaining 73.3% using albumin (79.3%), red blood cells (4.5%), or another crystalloid solution without heparin (16.2%). Hypotension occurred in 49.7% of the connections a median of 5 min after the beginning of the therapy. Fluid resuscitation was required in 38.5% and the dose of vasopressors was increased in 12.4% of the connections. There was no relationship between hypotension and age or weight. Re-priming the circuit with albumin reduced the incidence of hypotension from 71.4 to 44.6% (*p* = 0.004).

**Conclusions:**

Hypotension after the connection to CRRT is very frequent in critically ill children. Re-priming the circuit with albumin could improve hemodynamics during connection.

## Introduction

Acute kidney injury (AKI) is observed in up to 30% of the children admitted to a pediatric intensive care unit (PICU) [1]. Approximately 5% of the intensive care patients will be treated with renal replacement therapy. Continuous renal replacement therapy (CRRT) is most commonly used in this setting, since it is better tolerated than intermittent therapy from a hemodynamic point of view. Nevertheless, hypotension during and after the connection to CRRT is very common [2, 3]. In adult patients, hemodynamic instability during CRRT is associated with changes in circulating blood volume, blood flow rate variability, and electrolyte disturbances [4, 5]. In a prospective observational study of 1006 patients from 23 countries, Uchino et al. reported hemodynamic alterations related to CRRT such as hypotension in 19% and arrhythmic events in 4.3% of the patients [4]. Other investigators have reported hypotension after starting CRRT in 10–14% [6] and even 35% [7] of patients.

Children are at a higher risk than adults for developing complications associated with CRRT due to the large extracorporeal volume of the system (filters and lines) in proportion to their whole blood volume, which predisposes to hypotension at the time of connection. They require a more accurate control of volume status in order to avoid fluid and electrolyte disturbances. However, this complication has not been analyzed in depth in children. In a previous study of our group, hypotension during CRRT was found in 30.4% of the critically ill children [8].

There are a number of strategies that attempt to reduce the risk of hypotension at the time of connection, such as priming the circuit with whole blood or colloids but, as of yet, there are no comparative studies proving its efficacy over conventional priming solutions [9].

The objective of the present study was to analyze the process of CRRT connection, to determine the incidence of hypotension, and to analyze the predisposing risk factors.

## Methods

We conducted a single-center, longitudinal observational study of a cohort follow-up including critically ill children treated with CRRT between 2010 and 2014. Data were collected using a single-page case report form. Hemodynamic data (invasive arterial and venous blood pressure) and connection characteristics were collected before, during, and 60 min after the connection to CRRT. The circuit consisted of the Prismaflex® CRRT pump (Gambro, Barcelona, Spain) and either an AN69 polyacrylonitrile (M 10, M60 or M100) or a polyarylethersulfone (HF20) hemofilter set according to the age and weight of the patient: An M10 filter (Hospal, Lyon, France) was used in three patients weighing less than 10 kg at the beginning of the study period (surface 0.04 m^2^); an HF20 filter (Gambro Int.) for patients weighing less than 10 kg (surface 0.2 m^2^, total volume in set 60 ml); M60 filter (Hospal, Lyon, France) for patients between 10 and 35 kg (surface 0.6 m^2^, total volume in set 93 ml); and M100 filter (Hospal, Lyon, France) for patients weighing over 35 kg (surface 0.9 m^2^, total volume in set 152 ml). The caliber of the hemofiltration catheters depended on the age and weight of the patients, ranging between 4F and 11F. We used a blood warmer (Prismacomfort®; Gambro, Barcelona, Spain) in the return bloodline to compensate for heat loss to the atmosphere during the therapy.

A total dose of 50 UI/kg of heparin was administered pre-filter to all patients at the beginning of the therapy. When the circuit was re-primed with albumin, a bolus of 50 UI/kg of heparin was administered. When the circuit was not re-primed (heparinized normal saline was administered directly to the patient), the amount of heparin administered with the connection was subtracted from the total 50 UI/kg.

The following data were gathered prospectively in all patients upon starting CRRT: age, weight, sex, diagnosis, indication for CRRT, severity scores, number of affected organ systems (with dysfunction or failure), blood pressure, need for vasoactive drugs, dose of dopamine and adrenaline, lactic acid levels, type of hemofilter set and hemofiltration prescription variables and settings.

A specific CRRT connection protocol was not available in our PICU at the time of the study, so the way patients were connected to CRRT was determined by the physician in charge of the patient: some patients were connected after discarding the priming solution, some were connected directly using the original priming solution (normal saline with heparin), while other patients were connected after re-priming the circuit with 5% albumin. The investigator was present during the initiation of CRRT, recording the characteristics of the connection and the changes in hemodynamic parameters (heart rate, blood pressure, central venous pressure, vasopressor increase, and intravenous fluid bolus requirements) during the first hour of therapy. Hypotension prior to connection was defined as a mean arterial blood pressure under the 10th percentile according to age and height, and hypotension during connection was defined as a decrease in mean arterial pressure > 20% from baseline, the need for intravenous fluid expansion or the need to increase the dose of vasopressors during the first 60 min after the connection to CRRT.

STATA® statistical software version 13 (College Station, TX: StataCorp LP.9.2) was used for statistical analysis. Quantitative variables are expressed as means and standard deviations (SD) or as medians and interquartile range (IQR) (p25–p75), according to the normality or not of their distribution. Qualitative variables are expressed as percentages. The chi-square test, Fisher exact test, and Mann-Whitney test were used to compare qualitative and quantitative variables. Significance was taken as a *p* value less than 0.05. Wilcoxon rank test was used for the analysis of repeated measures.

## Results

During the study period, the characteristics of the connection to CRRT were studied in 36 children (27 boys (75%) and 9 girls (25%)) with a median age of 18.8 months (interquartile range (IQR) 5.2–84.0) and median weight of 9.95 kg (IQR 5.7–23.5). Table 1 shows the demographic characteristics of the patients included in the study: 41.7% of the patients were under 1 year of age and the most common underlying condition was heart disease, particularly during the postoperative period of cardiac surgery (28 patients, 77.8%). Most patients (94%) were on mechanical ventilation and 86.1% had vasopressors (median inotropic index was 46.5). The most common access and hemofiltration catheter were the femoral vein (78%) and a 6.5-Fr catheter (58%), respectively.

**Table 1:** Patient characteristics

	Median	IQR (p25–p75)
Age (months)	18.8	5.22–84.0
Weight (kg)	9.95	5.7–23.5
Body surface area (m^2^)	0.44	0.31–0.89
PRISM score (% mortality)	17.5 (21.3%)	11.5 (7.7%)–21.7(38.6%)
PIM score	18.1	9.0–30.0
Multiorganic failure score	3	3.0–4.0
P-MODS	6	5.0–8.0
PELOD score (% mortality)	21(20.8%)	11.0 (1.3%)–21.7(24.7%)
Lactic acid	1.9	0.9–4.5
Inotropic score	46.5	26.5–62
	Percentage	95% CI
Basal hypotension	20.5%	14–28%

*PRISM* pediatric risk of mortality, *PIM* pediatric index of mortality, *PELOD* pediatric logistic organ dysfunction, *P-MODS* pediatric multiple dysfunction score, *IQR* interquartile range, *95%CI* 95% confidence interval

**Fig. 1:**
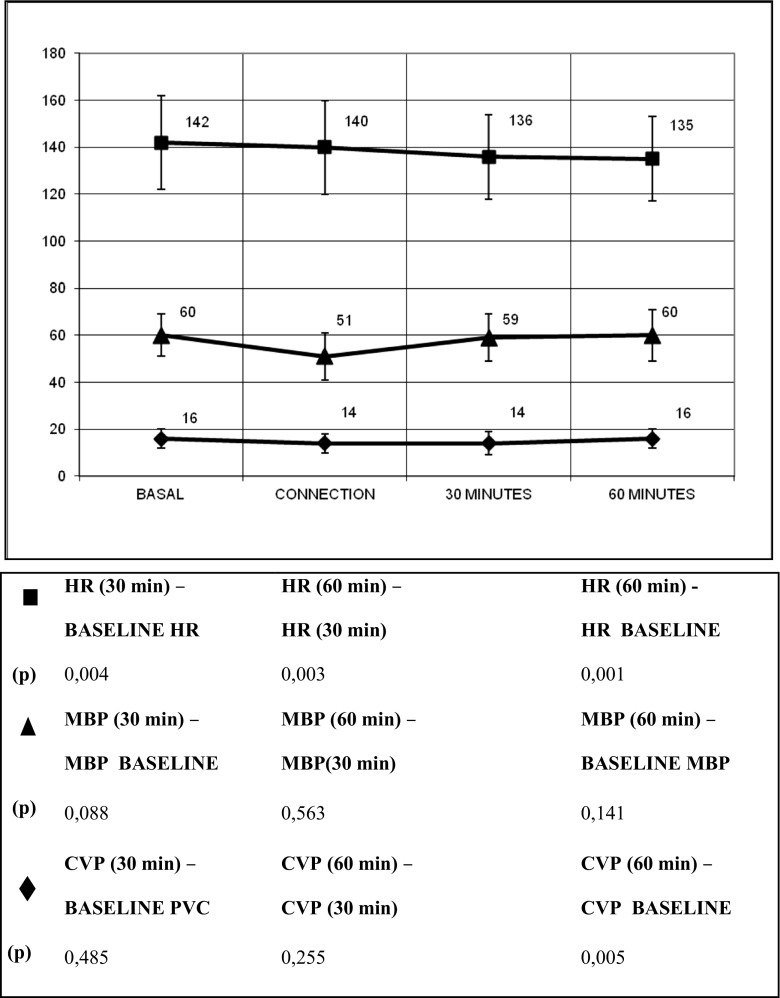
Evolution of hemodynamic parameters during the first hour after connection. *CVP* central venous pressure, *HR* heart rate, *MBP* mean blood pressure. Median and standard deviation

A total of 161 circuit connections were analyzed. The most commonly used filter was the 0.2-m^2^ surface filter (47%), followed by the 0.6-m^2^ surface filter (45%), and the 0.9-m^2^ filter (8%). Median blood flow at the time of connection was 5 ml/kg/min (IQR 3–6 ml/kg/min). The priming solution was discarded in 8.7% of the connections, the heparinized priming solution was infused in 18% of the connections, and the circuit was re-primed with albumin 5% or crystalloid solutions (0.9%) without heparin in 73.3% of the cases. Figure 1 shows the evolution of hemodynamic parameters at baseline (before the connection), at connection, at 30 min, and at 60 min after the connection. Mean blood pressure (MAP) and central venous pressure (CVP) dropped significantly at connection (*p* < 0.01) without any significant changes in heart rate. Hypotension was recorded in 49.7% of the connections. Lowest mean arterial pressure was registered at a median of 5 min after initiating CRRT. Fluid resuscitation was required in 38.5% of the connections (median fluid volume 5.2 ml/kg) and the dose of inotropes had to be increased in 12.4% (median increment of the vasoactive-inotropic score from 41 to 52 points). All parameters except heart rate returned to baseline at 30 and 60 min of therapy. Heart rate slowly and progressively decreased throughout the first hour of therapy.

Table 2 compares baseline parameters between hypotension and no-hypotensive episodes during the connection to CRRT.

**Table 2:** Comparison between hypotensive and non-hypotensive episodes

Variable	Hypotension Mean (SD)	No Hypotension Mean (SD)	*p*
MAP at baseline	59 (SD 9)	62 (SD 10)	0.18
HR at baseline	142 (SD 21)	140 (SD 19)	0.61
CVP at baseline	16 (SD 5)	15 (SD 5)	0.23
Lactic acid	2.6 (SD 2.5)	2.7 (SD 2.2)	0.88
Vasopressor-inotropic score at baseline	52 (SD 37)	39 (SD 39)	0.45
Filter surface/BSA	1 (SD 0.5–1.6)	0.6 (SD 0.5–1)	0.008
Blood flow (ml/kg/min)	5.5 (SD 2.6)	5.9 (SD 2.4)	0.04

*MAP* mean arterial pressure, *HR* heart rate, *CVP* central venous pressure, *BSA* body surface area, *SD* standard deviation

There was no relationship between hypotension and age or weight (Table 3) but the quotient filter-surface/body surface area (BSA) was significantly higher in patients who developed hypotension (median 1, IQR 0.5–1.6) than in those who did not (median 0.6, IQR 0.5–1), *p* = 0.008.

**Table 3:** Factors related with hypotension during connection

Variable	Group	Number	Percentage of hypotension (%)	Significance (*p*)
Age (months)	< 12 months	80	50.7	0.446
	> 12 months	81	53.1	
Weight (kg)	< 10 kg	102	46.3	0.097
	> 10 kg	59	61	
Filter Surface/BSA > 0.8	≥ 0.8	72	62.5	< 0.01
	< 0.8	89	38.2	
MAP < p10 at baseline	Yes	33	65.6	0.111
	No	128	47.9	
Vasopressor-inotropic score at baseline	> 40	87	76.9	0.659
	< 40	74	63.6	
Re-priming	Yes	118	44.6	0.004
	No	43	71.4	
Re-priming solution	Crystalloid	47	58.7	0.007
	Colloid	93	41.6	
Study year	2010–2011	51	70	0.001
	2012–2014	110	43.3	

*MAP* median arterial pressure, *p10* 10 percentile, *BSA* body surface area

There were no differences between hemodynamic parameters at baseline (Table 2).

We did not find a relationship between hypotension during connection and severity illness scores or lactic acid levels either. Nevertheless, patients with a higher vasopressor-inotropic score and lower mean arterial pressure MAP prior to CRRT initiation had a greater (although not statistically significant) incidence of hypotension (Table 3).

Re-priming of the circuit before the connection reduced the incidence of hypotension from 71.4 to 44.6% (*p* = 0.004). The incidence of hypotension was higher in circuits that were re-primed with crystalloids than with albumin (58.7% vs 41.6%, *p* = 0.07). We also observed that after the first 2 years of the study, the incidence of hypotension during connection significantly decreased from 70 to 43.3% (*p* = 0.001).

## Discussion

Hypotension is a common complication at the beginning of CRRT in hemodynamically unstable patients. In a worldwide survey in adults, hypotension was observed in 18.8% of the patients [4]. Children are at a higher risk than adults for developing hypotension at the time of connection due to the large extracorporeal volume of the system that includes filters and lines. In our previous study on complications during CRRT, hypotension was found in 30.4% of the children, but we did not specifically analyze the characteristics of the connection [8]. The type of priming solution was not recorded, so it was not possible to determine whether priming with a colloid was associated with a better hemodynamic performance in terms of less need for volume resuscitation or changes in the dose of vasoactive drugs. The aim of this present study was to analyze the characteristics of the connection process in terms of CRRT variables and settings as well as the type of priming solution and its repercussion on hemodynamics in order to develop and to implement a specific CRRT connection protocol in our unit.

The patients included in this study were severely ill, as 94% of them were on mechanical ventilation and had high mortality scores and 86.1% had vasopressors (median vasopressor-inotropic index of 46.5). Hypotension prior to the beginning of CRRT was observed in 20.5% of the cases. It is to be expected that patients with greater hemodynamic alterations at baseline would be at a higher risk for developing hypotension when connecting to CRRT. In our study, hypotensive patients became even more hypotensive during connection in 65.6% of the cases, whereas the incidence of hypotension upon connection in previously non-hypotensive patients was 47.9%. This difference, however, did not reach statistical significance. Global incidence of hypotension in our study was 49.7% of the connections, a median of 5 min after the beginning of the therapy. This incidence is even higher than that reported in our previous study, which was 30.4% [8], which is probably due to a more exhaustive observation of hemodynamic changes during the first minutes of the connection.

We did not find any relationship between hypotension and age or weight. Interestingly, the quotient filter-surface/BSA was significantly higher in patients who developed hypotension. This fact highlights the relevance that the priming volume of the set has on hemodynamics during connection, as it can represent up to 5 to 10% of the patients’ total blood volume [10]. The priming volume of the circuits that were used in the study ranged from 60 to 152 ml. It is of vital importance to enhance the design of hemofilter sets with the lowest possible priming volume in order to reduce hemodynamic repercussions when connecting pediatric patients to CRRT.

No individual risk factors for hypotension were found in this study. Nevertheless, it is possible that different individual factors combined together (such as the extracorporeal volume of the circuit and previous hemodynamic alterations) could add up to the risk of developing hypotension after connection to CRRT.

In 2012, Eastwood et al. [3] analyzed the hemodynamic impact of the blood pump speed at the beginning of CRRT in critically ill adults, comparing “routine-protocol” pump speed increases of 50 ml/min over 1–4 min with “slower” increases of 20–50 ml/min over 3–10 min. They observed significant differences between both groups only in heart rate, but not in blood pressure. However, they recommend that close hemodynamic monitoring should be performed during connection. It is interesting to note that the use of albumin 5% for re-priming the circuit increased throughout their study.

Nevertheless, that same group [2] published a retrospective study to analyze the impact of the slow blood flow protocol in vasopressor-dependent patients. They studied 205 circuit connections and a decrease in MAP > 20% was only observed in 16 out of 205 connections (7.8%) and CRRT did not have to be discontinued because of hypotension in any patient.

We did not have a specific “CRRT connection protocol” at the time of the study. A slow CRRT connection protocol was introduced afterwards, in the light of the results of the present study. During the first years of the study, the priming volume was discarded in most cases (14 connections) to avoid administering a high dose of heparin to the patient. Only the most unstable patients were connected after re-priming the circuit with crystalloids or colloids without heparin. The incidence of hypotension in this study period was very high (71.4%). After the first 2 years of the study, we decided not to discard the priming solution and encouraged physicians to re-prime the circuit with albumin 5%. The incidence of hypotension decreased significantly in this period from 71.4 to 44.6% (*p* = 0.004).

The difference in the incidence of hypotension between colloids and crystalloids were almost statistically significant (58.7% vs 41.6% (*p* = 0.07)). Further studies are necessary to determine the efficacy of re-priming the circuit with albumin in reducing the incidence of hypotension at the time of connection.

The way hypotension was managed differed with each patient’s situation. The first step was usually volume resuscitation with 10 to 20 ml/kg of a colloid solution. The median volume that was necessary to restore blood pressure was 5 ml/kg. Next, if hypotension was severe or if it did not respond to volume resuscitation, the dose of vasoactive drugs was increased. The median increase in vasoactive-inotropic score to restore hemodynamic stability in our study was 11 points. Some patients could benefit from a preventive increase in vasopressors prior to connection to avoid or minimize hypotension.

Excessive ultrafiltration is also a risk factor for hypotension [5]. To prevent this, we did not program ultrafiltration or negative fluid balance during the connection to CRRT, until the hemodynamic situation was stabilized.

## Limitations

Our study has several limitations. In the first place, the way the patient was connected to CRRT depended on the physician in charge of the patient. The results are a good reflection of the clinical practice at that moment, but could be biased due to the personal preferences of each physician. Another limitation is that we only analyzed initial blood flow during connection, but not the speed at which the blood flow was increased.

## Conclusions

Hypotension after the connection to CRRT is frequent in critically ill children. For this reason, close hemodynamic monitoring during connection is necessary. Re-priming the circuit with albumin 5% could improve hemodynamics during connection. Further studies are needed to determine the ideal priming solution and the relevance of a slow blood flow protocol.
